# The increased adhesion of tumor cells to endothelial cells after irradiation can be reduced by FAK-inhibition

**DOI:** 10.1186/s13014-019-1230-3

**Published:** 2019-02-04

**Authors:** Pascaline Nguemgo Kouam, Helmut Bühler, Thomas Hero, Irenäus A. Adamietz

**Affiliations:** 1 Institute for Molecular Oncology, Radio-Biology and Experimental Radiotherapy, Universitätsklinikum Marien Hospital Herne, Ruhr-Universität Bochum, Herne, Germany; 2 Department of Radiotherapy and Radio-Oncology, Universitätsklinikum Marien Hospital Herne, Ruhr-Universität Bochum, Herne, Germany; 3 Universitätsklinikum Marien Hospital, Hölkeskampring 40, 44265 Herne, Germany

**Keywords:** Ionizing radiation, Adhesion, Adhesion-associated protein, FAK-inhibitor

## Abstract

**Background:**

Radiotherapy is administered in more than 60% of all solid tumors. Most patients are cured but a significant number develops local recurrences or distant metastases. The question arises if irradiation might influence the metastatic process. In the present study we examined whether the adhesion of glioblastoma or breast cancer cells to endothelial cells, an important step in metastasis, is affected by photon irradiation.

**Methods:**

U-87 MG, U-373 MG and MDA-MB-231 cancer cells as well as primary human endothelial cells were irradiated with 0, 2, 4, or 8 Gy photons at a dose rate of 5 Gy/min. The adhesion of cancer cells to endothelial cells was tested either with the Vybrant based assay via fluorescent labelling or with an ibidi pump system able to mimic the physiological blood flow in vitro. In addition, the impact of FAK (focal adhesion kinase) inhibitor PF-573, 228 on the adhesion of non-irradiated and irradiated tumor cells was analyzed. Adhesion related and regulated proteins were analyzed by Western blotting.

**Results:**

The cellular adhesion was increased after irradiation regardless of which cell type was irradiated. The FAK-inhibitor was able to reduce the adhesion of non-irradiated cells but also the irradiation-induced increase in adhesion of tumor cells to endothelium. Adhesion related proteins were enhanced after irradiation with 4 Gy or 8 Gy in both cells types. The increased adhesion after irradiation is accompanied by the phosphorylation of src (Y416), FAK (Y397) and increased expression of paxillin.

**Conclusion:**

Irradiation with photons in therapeutic doses is able to enhance the interaction between tumor cells and endothelial cells and by that might influence important steps of the metastatic process.

## Background

Radiation is part of standard therapies in clinical oncology due to its effective local tumor control and curative potential for many cancer types [1, 2]. However, there were various observations in the earliest stages of radiation oncology that ineffective irradiation of solid tumors could ultimately result in the enhancement of metastasis [3–7]. It is conceivable that the surviving cells, tumor cells as well as neighboring cells such as endothelial cells are modified phenotypically and genetically by irradiation. There is evidence in the literature that such surviving radiation-resistant tumor cells metastasize more frequently in animal models and could also lead to recurrence in patients [8–12]. Every new formation of tumors – whether as local recurrence, as micrometastasis in the bone marrow or as distant metastasis – results from an interaction of the tumor cells with their environment [9, 13–15]. Of particular importance here are the blood and lymph vessels [16]. During the process of metastasis, the tumor cell first interacts with the endothelium, which slows down its speed (rolling) until it adheres locally (adhesion). Only then can the tumor cell leave the vessel and colonize new tissue [17, 18]. Adhesion is therefore a decisive step in metastasis. In a similar way to the migration of leukocytes from the vessel in response to inflammatory processes, the adhesion of tumor cells is regulated by certain surface molecules. This adhesion can take place directly between tumor cells and endothelium with the help of adhesion-associated proteins or indirectly via leukocytes [19, 20]. However, it remains largely unclear how irradiation affects this cell-cell interaction and how the adhesion-associated proteins change. We have therefore used various macroscopic methods to investigate whether and how irradiation with photons alters the adhesion of breast cancer cells or glioblastoma cells to an endothelial cell monolayer and whether this is reflected in the expression of associated proteins. Breast cancer is one of the of most common tumor types that is well known to form local recurrences and metastases. Approximately 10% of all breast cancer patients have already developed verifiable distant metastases at the initial diagnosis and post-therapeutic metastases are not unusual [21]. In contrast, 90% of cases of glioblastomas are characterized by local recurrences [22, 23]. Nevertheless, both loco-regional and distant metastases are observed in some cases [24]. Radiotherapy is commonly used as a treatment method for both tumor types. The differences in the metastatic properties of these two tumor entities could be of great clinical relevance with respect to whether irradiation affects adhesion of circulating tumor cells to the vascular endothelium. Special attention was also paid to focal adhesion kinase (FAK), which plays an important role in the regulation of integrin signaling, cell adhesion, migration and proliferation of cells. Decisive steps here are the autophosphorylation of FAK and its complex formation with paxillin and Src [25–27]. In many tumor entities such as glioblastoma and breast cancer, FAK is often overexpressed, which correlates with increasing tumor malignancy [28]. In addition to irradiation, we investigated the influence of an additional inhibition of FAK with the inhibitor PF-573, 228. This inhibitor inhibits phosphorylation and thus activation of FAK and its downstream effector paxillin and thus influences migration and adhesion [29–33]. Our data show an increase in the adhesion of tumor cells to the endothelial cells after irradiation, which correlates with an upregulation of adhesion proteins. Phosphorylation of Src (Y416) and FAK (Y397) seems to play an important role and may potentially be inhibited by the inhibitor PF-573, 228, similarly to increased adhesion.

## Materials and methods

### Cell culture

The glioblastoma cell lines used, U-87 MG and U-373 MG, and the breast cancer cell line MDA-MB-231 were purchased from the American Type Culture Collectio*n* (ATCC, Manassas, VA, USA). The cells were cultivated in DMEM (Dulbeccos’s modified Eagle medium), supplemented with 10% fetal calf serum (FCS), 100 U/ml penicillin and 100 μg/ml streptomycin (Biochrom, Berlin, Germany) in the incubator at a temperature of 37 °C and with 5% CO_2_ in the air. Primary HUVEC (human umbilical vein endothelial cell) cells (Cat. #C-12206) (PromoCell, Heidelberg, Germany) were cultivated in Endopan medium (Cat. #P0a-0010 K) (PAN-Biotech, Aidenbach, Germany) under the above-mentioned conditions. For the experiments HUVEC cells were used which had been passaged between 4 and 6 times. For the experiments, frozen low-passage cells were taken into culture. The authenticity of the cells was ensured by morphology, expression of lead proteins, proliferation and migration parameters. In particular, it was ensured that the U373 cells used were not U251 cells, as the literature suggests that there had been confusion at cell banks. A mycoplasma test was performed regularly (approx. 5 times per year).

### Irradiation

HUVEC cells and tumor cells were irradiated at room temperature with doses of 0, 2, 4, or 8 Gy photons at a linear accelerator (Synergy S, Elekta, Hamburg, Germany), at 6 MeV and a dose rate of 5 Gy/min.

### Incubations with the inhibitor PF-573, 228

This substance is of low solubility in water and was therefore added to the cell culture medium from DMSO stock solutions. The proportion of DMSO in the culture medium was 0.1%, a concentration that does not impair cell vitality. For untreated controls, DMSO was added alone.

### Proliferation test and treatment of cells with PF-573, 228

On a 96-well plate 5000 cells per well were seeded in 100 μl medium and cultivated for 24 h at 37 °C and 5% CO_2_. On the next day, various concentrations of the PF-573, 228 inhibitor (Cat. No. 3239, Tocris Bioscience, USA) (0; 0.001; 0.01; 0.1; 1; 10; 100 μM) were added to the cells. After 24 h, 48 h and 72 h incubation, 25 μl of a 5 mg/ml MTT solution were added to the cells and incubated for 2 h. The formazan crystals formed from MTT were solubilized for 30 min at 37 °C by adding 100 μl stop solution (99.4 ml DMSO, 10 g SDS and 0.6 ml acetic acid). Subsequently, the relative proliferation rate was determined by measuring the extinction at 570 nm in an ELISA reader (TECAN infinite 200 M).

### Adhesion assay using calcein fluorescence labelling

For the adhesion test, the tumor cells were cultured in a T25 cm^2^ culture flask up to approx. 80% confluency. The tumor cells were treated with 1 μM PF-573, 228 inhibitor 24 h before irradiation. 60 min before irradiation, the substance was removed, the cells were washed with PBS and the medium was replaced. Controls without inhibitor were treated in the same way. 15,000 primary HUVEC cells per well were seeded on a 96-well plate and cultured at 37 °C and 5% CO_2_ until the cells were fully confluent. After irradiation, the tumor cells were incubated in the incubator for 30 min before being used for the experiment. Then the medium was aspirated, the cells were washed twice with PBS and removed with trypsin. The cells were then suspended and incubated with calcein (1 mM) for 30 min at 37 °C and 5% CO_2_ in a 50 ml tube. In between, the tube was carefully swiveled to ensure a homogeneous staining of all cells. After staining, the cells were washed three times with PBS and 50,000 cells each were placed in 100 μl medium on the endothelial monolayer and incubated for 4 h at 37 °C and 5% CO_2_. After incubation, the first measurement was taken at 495 nm / 540 nm in the ELISA Reader. The unattached cells were then carefully aspirated, and the wells were washed three times with 400 μl PBS and measured a second time in the ELISA Reader. The ratio between the two measurements was then used to determine the quantity of adhered cells.

### Adhesion assay using the IBIDI pump

The IBIDI pump system (Fluidic Unit: Cat.No. 10903; Perfusion set (50 cm, ID 1.6 mm): Cat No. 10964; μ-slide I^0.6^ Luer: Cat. No. 80186. ibidi GmbH, Munich, Germany) is well-suited for the cultivation of cells under flow conditions to simulate blood vessels. In this experiment, a continuous flow was generated. The pump system was constructed according to the instructions (IBIDI GmbH, AN 13: HUVECs under Perfusion). 250,000 primary HUVEC cells were seeded onto a μ-slide I^0.6^ Luer and incubated for 24 h at 37 °C and 5% CO_2_ in the incubator to form a monolayer. The next day, tumor cells were stained with calcein as described above. A 12 ml tumor cell suspension with 30,000 cells / ml was placed in the reservoir of the μ-Slide. The experiment was conducted under the following conditions: 6 mbar pressure, 2.5 ml/min flow rate and a shear stress of 1.9 dyn/cm^2^. With the help of our self-built video microscopy system [34], the circulating cells in the μ-slide Luer were observed. After completion of the experiment, all attached tumor cells were counted in three visual fields.

### Protein isolation and immunoblot analysis

To isolate proteins from monolayer cell cultures, medium was aspirated, cells washed with PBS, and subsequently lysed in hot 1x Roti-Load sample buffer (Carl Roth, Karlsruhe, Germany) with additional homogenization using an ultrasonic probe (Misonix, Farmingdale, NY, USA). Lysates were incubated at 90 °C for 5 min and cleared by centrifugation (1 min, 10,000 g). 15 μl of the protein lysates were separated using (8–10%) SDS-PAGE and blotted onto nitrocellulose membranes (Schleicher & Schüll, Dassel, Germany) in a tank blot unit (Mini-PROTEAN II, BioRad, Hercules, CA, USA). After blocking with a 3% BSA solution, membranes were incubated with primary antibodies reacting with: E-selectin (Cat. PA5–29946, Thermo Scientific, USA), paxillin (1:1000: Cat. #MS-404-P0, NeoMarkers, USA; 1:500: Cat #2542, Cell Signaling Technology), integrin β1 (1:1000: Cat. #9699), integrin α4 (1:1000: Cat. #4600), integrin α5 (1:500; Cat #4711), N-cadherin (1:1000; Cat. #4061), ICAM-1 (1:500: Cat. #4915), VCAM-1 (1:1000: Cat. #12367), CD44 (1:1000: Cat. #5640), focal adhesion kinase (FAK) (1:1000: Cat. #3285), phospho-FAK (Tyr 925) (1.1000: Cat. #3284), phospho-FAK (Tyr 397) (1.1000: Cat. #3283), Src (1:1000: Cat. #2109), phospho-Src (Tyr 416) (1:500: Cat. #2101) (Cell Signaling Technology, Frankfurt, Germany), and β-actin-POD (1:25,000: Cat. #A3854, Sigma-Aldrich, Taufkirchen, Germany). HRP-conjugated secondary antibodies (anti rabbit HRP (1:1000) and anti mouse (1:1000); Thermo Scientific, USA) and the Lumi-Light plus Western Blotting Substrate (Roche Diagnostics, Mannheim, Germany) were used. Chemiluminescence was recorded using the ChemiDoc MP system and Image Lab program (Bio-Rad, Munich, Germany).

### Statistical analysis

GraphPad Prism (GraphPad Software, La Jolla, CA) was used for data analysis (Student’s t-test).

## Results

### Irradiation promotes the adhesion of tumor cells to the endothelial monolayer in vitro

**Fig. 1:**
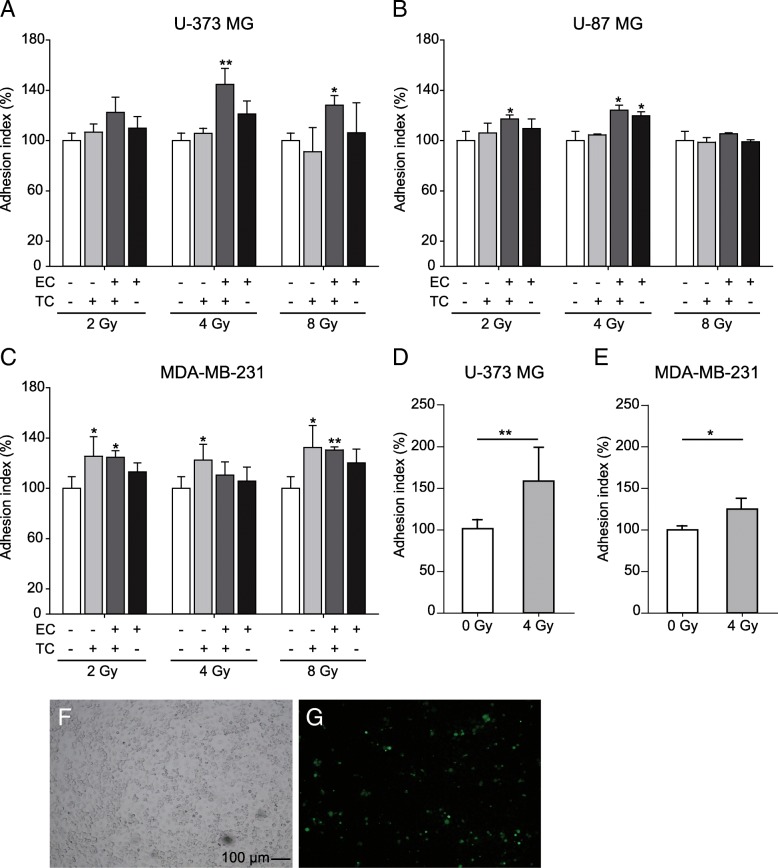
Influence of irradiation on adhesion of tumor cells to an endothelial cell monolayer. **a, b, c** Adhesion of glioblastoma cells (U-373 MG, U-87 MG) and breast cancer cells (MDA-MB-231). Tumor cells and primary HUVEC were irradiated with 2 Gy, 4 Gy or 8 Gy. The tumor cells were loaded with calcein, placed on an endothelial cell monolayer, incubated for 4 h and then washed out. The number of adherent tumor cells was determined by measuring the remaining calcein fluorescence. The determined adhesion index was normalized to the adhesion index of non-irradiated TC on non-irradiated EC (100%) (*n* = 8). TC: tumor cells, EC: endothelial cells. **d, e** Measurement of adhesion of U 373 MG and MDA-MB-231 cells under flow (*n* ≥ 3). 30,000 tumor cells/ml irradiated with 4 Gy circulated for 2 h at room temperature over a non-irradiated endothelial monolayer. The number of adherent tumor cells was counted microscopically in three visual fields. Mean values ± SEM are shown. Statistical significance was determined with the Student’s t test: *p < 0.05; **p < 0.01. **f, g** Representative image of adherent tumor cells (U-373 MG) in the flow chamber. Tumor cells were labeled with calcein (**g**)

Irradiation of tumor cells and endothelial cells significantly increases adhesion (Fig. 1). Adhesion of U-87 MG and U-373 MG glioblastoma cells to non-irradiated endothelial cells increased by approximately 5 to 10% after irradiation with 2, 4, and 8 Gy. An increase of about 10 to 15% resulted when non-irradiated glioblastoma cells were applied to endothelial cells irradiated with 2 or 4 Gy. In contrast, a dose of 8 Gy did not induce any change (Fig. 1a, b). Irradiation of both cell types (endothelial cells and glioblastoma cells) caused the highest increase in adhesion. Here, for example, the adhesion of U-87 MG with a radiation dose of 4 Gy increased by approx. 25% and of U-373 MG by 42% (Fig. 1b). In addition, we investigated the adhesion of irradiated circulating tumor cells to the endothelial monolayer in the flow chamber. For this purpose, irradiated tumor cells (4 Gy) were circulated under physiological flow conditions over non-irradiated endothelial monolayers and then the number of adhered tumor cells was counted. After irradiation, approximately 45% more adherent tumor cells were counted than in non-irradiated controls (Fig. 1d). We also applied the static as well as the flow chamber adhesion assay to breast cancer cells, in order to investigate the influence of irradiation on adhesion in an additional and different tumor entity. As with the glioblastoma cells, increased adhesion of breast carcinoma cells (MDA-MB-231) to the endothelial monolayer was measured (Fig. 1c, e). Here, irradiation of MDA-MB-231 cells with 2 Gy, 4 Gy and 8 Gy led to a 25, 22 and 30% increase in adhesion, respectively. Increased adhesion of 16 to 30% was also found after irradiation of both MDA-MB-231 cells and HUVEC. This observed increase was significant but weaker than after irradiation of MDA-MB-231 cells alone. Irradiation of endothelial cells alone did not lead to a significant change in adhesion of non-irradiated MDA-MB-231 cells (Fig. 1c). In the flow chamber 25% more adherent tumor cells were counted after the irradiation with 4 Gy (Fig. 1e).

### Several adhesion-associated proteins examined were upregulated after irradiation

**Fig. 2:**
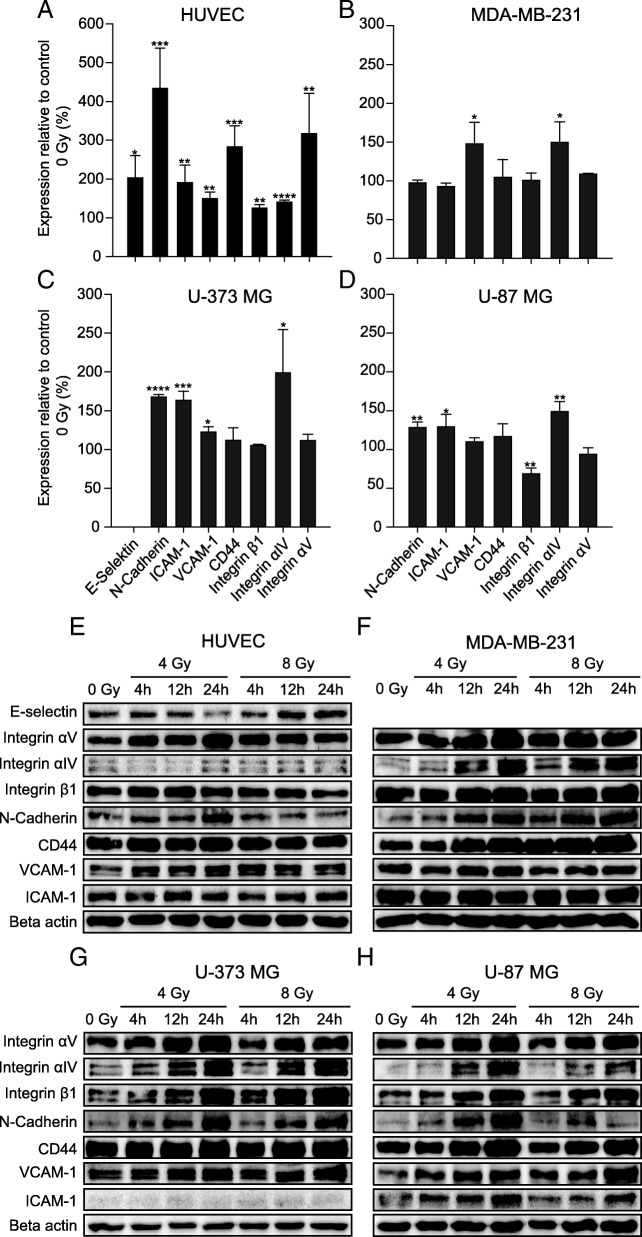
Influence of irradiation on the expression of adhesion proteins. Western Blot analysis with samples from primary HUVEC (**a, e**), breast cancer cells (MDA-MB-231 (**b, f**) and glioblastoma cells (U-373 MG (**c, g**) and U-87 MG (**d, h**)). *Above*: **a - d**) The sample was isolated 4 h after irradiation with 4 Gy. What is shown is the average value normalized to non-irradiated (0 Gy) values. Data represent means ± standard deviation (SD) from at least three separate experiments (n ≥ 3). *p < 0.05; **p < 0.01; ***p < 0.001 (Student’s t test). *Below*: **e - h**) Examples of one single experiment each. Protein expression in Western blots 4, 12 and 24 h after irradiation with 4 Gy or 8 Gy

The increased interaction between tumor cells and endothelial cells after irradiation could be due to a change in the composition of the surface proteins involved in the adhesion processes. We therefore analyzed the expression of a number of adhesion-associated proteins (E-selectin, N-cadherin, ICAM-1, VCAM-1; CD44, integrin α4, integrin α5, integrin β1) in tumor cells and endothelial cells using Western blot (Fig. 2). In both endothelial cells and tumor cells, irradiation with 4 Gy after 4 h led to an increase in the expression of almost all proteins investigated. In endothelial cells an increase in protein expression between 25 and 200% was found (Fig. 2a). In tumor cells the expression rose by an average of 20% (Fig. 2b - d). Protein expression remained elevated 12 h and 24 h after irradiation with 4 Gy and also with 8 Gy (Fig. 2e - h).

**Fig. 3:**
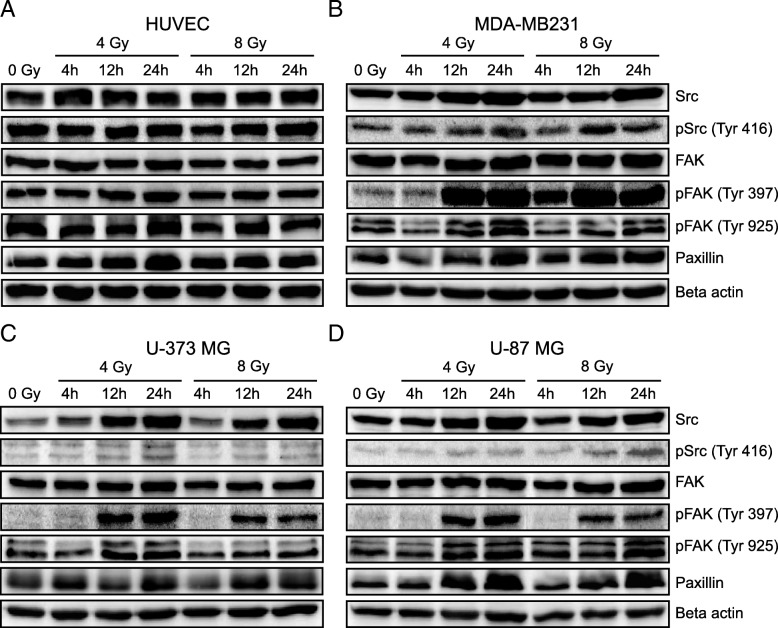
Influence of irradiation on the expression of adhesion-regulating proteins. **a - d** Examples of Western blot analysis of adhesion-regulating proteins in breast cancer cells (MDA-MB-231), glioblastoma cells (U-373 MG and U-87 MG) and protein expression in primary HUVEC cells. Cells were irradiated with 4 Gy or 8 Gy and protein isolation was performed 4 h, 12 h and 24 h after irradiation

Furthermore, it was investigated whether irradiation also influences the expression and activation of proteins involved in the regulation of adhesion and migration processes (Src, phopho-Src-Y416, FAK, phospho-FAK-Y925, phospho-FAK-Y397 and paxillin). Both expression and activation were upregulated after irradiation and remained high for hours (Fig. 3). In endothelial cells the expression of FAK hardly changed after irradiation with 8 Gy, while the expression of Src and its phosphorylation on tyrosine 416 increased independently of the radiation dose. The phosphorylation of FAK on tyrosine 397, 925 and the expression of paxillin also increased (Fig. 3a). In tumor cells, the effect of radiation was much stronger. Some adhesion-regulating proteins studied increased significantly. Src and phospho-Src-Y416 almost doubled after irradiation depending on tumor cell line. The phosphorylation of FAK on tyrosine 925 only increased significantly 12 h after irradiation. The same result was observed in the expression of paxillin. Expression only increased clearly after 12 h. The increase of phospho-FAK-Y397 in irradiated tumor cells was also remarkable. Here, irradiation induced the phosphorylation of FAK on tyrosine 397, which increased over time (Fig. 3b – d). Radiation appears to trigger phosphorylation of Src on tyrosine 416 and FAK on tyrosine 397 primarily in tumor cells. It is noticeable that HUVEC, in contrast to the tumor cells, already show a strong phosphorylation of FAK without irradiation and the relative increase after irradiation is therefore weaker.

### FAK-inhibition reduces the irradiation-induced adhesion

**Fig. 4:**
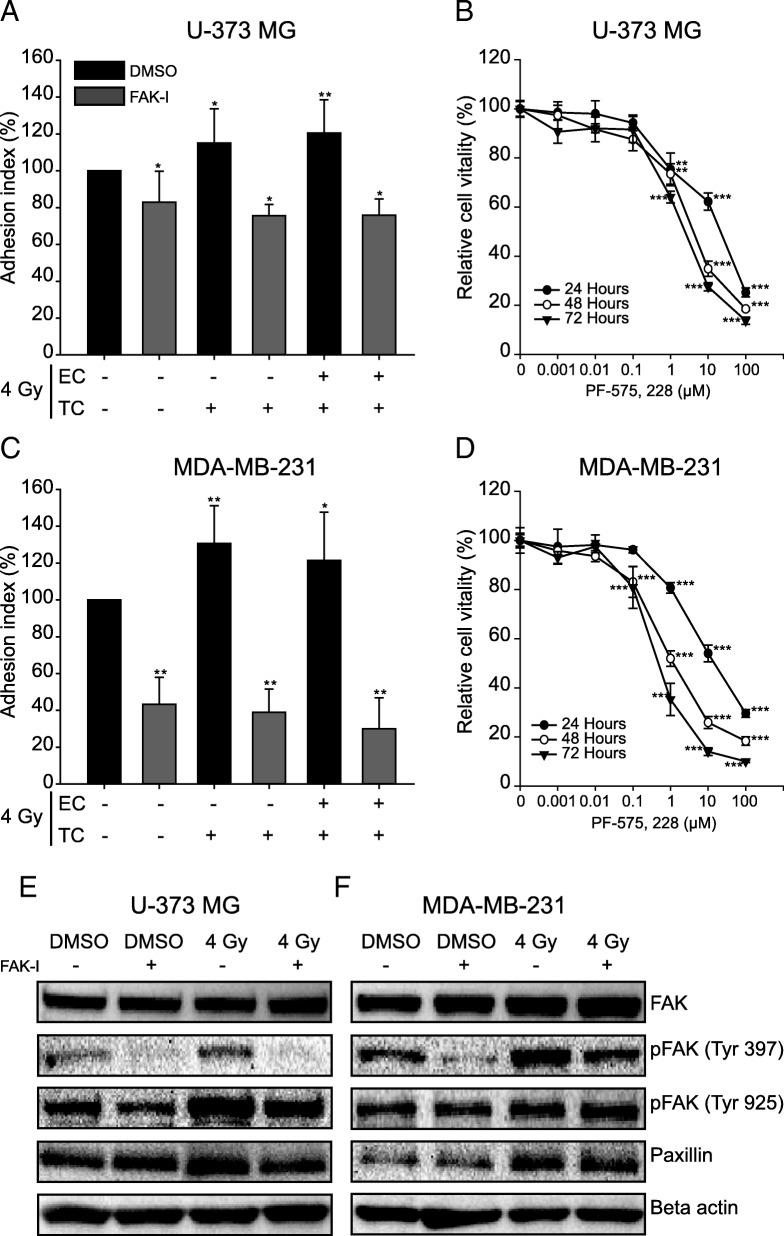
Inhibition of tumor cell adhesion by the PF-573, 228 inhibitor. **a, c** Adhesion test. The tumor cells were incubated with 1 μM PF-573, 228 inhibitor or vehicle alone (0.1% DMSO) for 24 h before irradiation with 4 Gy. The fluorescence-labelled tumor cells were placed on a monolayer of primary HUVEC cells and incubated for 4 h. After washing out the non-adherent tumor cells, the adhesion rate was quantified in the fluorescence reader and normalized (100%) to the adhesion of untreated cells (TC and EC). **b, d** Cell proliferation and determination of the toxic effect of the PF-573, 228 inhibitor (FAK-I) with a MTT viability test. Cells were treated with different concentrations of the inhibitor for 24 h, 48 h and 72 h. The control cells were treated with DMSO (0.1%) alone. Shown are means (*n* = 3) and standard deviations. Statistics: T-test, *p < 0.05; **p < 0.01; ***p < 0.001. **e, f** Western blot analysis of the impact of this inhibitor on the phosphorylation of FAK and its downstream target paxillin in U-373 MG cells (**e**) and MDA-MB-231 cells (**f**). The samples were isolated 24 h after irradiation with 4 Gy and treatment with 1 μM PF-573, 228

Next, it was investigated whether the inhibition of phosphorylation and thus of the activation of FAK in tumor cells could reduce the interaction between tumor cells and endothelial cells. First, it was examined whether the FAK inhibitor PF-573, 228 influences the proliferation of tumor cells in a dose-dependent manner. The proliferation rate was determined after 24, 48 and 72 h for concentrations between 0.001 and 100 μM (Fig. 4b, d). For inhibitor concentrations up to 0.1 μM, no significant influence on the proliferation rate was observed in either glioblastoma or breast cancer cells, regardless of incubation time. From 1 μM upwards the proliferation decreased depending on the dose with stronger effects during longer incubation. For the adhesion test in the presence of the inhibitor, a concentration of 1 μM was chosen at which an inhibition of proliferation of approx. 20–25% was measured (Fig. 4b, d).

Treatment of glioblastoma cells U-373 MG with 1 μM PF-573, 228 inhibitor reduced adhesion of both non-irradiated and irradiated cells. In the non-irradiated tumor cells, a reduction in adhesion of approx. 20% was observed, which was even slightly stronger after irradiation. The radiation-induced increase in adhesion was thus completely prevented by the inhibitor (Fig. 4a). In breast cancer cells MDA-MB-231, the same but significantly stronger effects were observed. After irradiation with 4 Gy without inhibitor, 20 to 25% more MDA-MB-231 cells adhered to the endothelial cells. The inhibitor reduced the adhesion of both non-irradiated MDA-MB-231 cells and irradiated cells by 50 to 60% (Fig. 4c). The impact of this inhibitor on the phosphorylation of FAK and on the expression of its downstream effector paxillin in tumor cells was than investigated. PF-573, 228 inhibitor did not affect the expression of FAK (Fig. 4e, f). Treatment of glioblastoma cells with this inhibitor reduced the irradiation-induced increase in phosphorylation of FAK on tyrosine 925 and suppressed the phosphorylation on tyrosine 397 even after irradiation (Fig. 4e). In contrast to glioblastoma cells, the inhibition of FAK in breast cancer cells had no effect on the phosphorylation of FAK on tyrosine 925 and the phosphorylation on tyrosine 397 was just partially suppressed (Fig. 4f). With regard to paxillin, the inhibitor slightly reduced the radiation-induced increase in expression. It had no effect on expression in non-irradiated cells.

## Discussion

In addition to its antiproliferative and cytotoxic effects, radiation causes many changes that can be measured at both the cellular and molecular level. These effects are not only limited to the cells in the irradiation field, but can also be observed in neighboring cells [35]. Our investigations show that radiation increases the expression of adhesion-associated proteins both in tumor cells and in endothelial cells and thus increases the adhesion of tumor cells to an endothelial monolayer. We observe 5 to 20% more adhesion of tumor cells to primary HUVEC cells after irradiation with both 2 Gy and 4 Gy (Fig. 1a - c). Nübel et al. showed for the first time that ionizing radiation increased the adhesion of tumor cells to endothelial cells. They incubated non-irradiated colon cancer cells for five hours with primary HUVEC cells that had received different therapeutic radiation doses. They observed that the increase in the expression of E-selectin under irradiation was associated with an increase in the adhesion of tumor cells [36]. Our investigations also showed an increase in the adhesion of non-irradiated tumor cells to an irradiated primary HUVEC monolayer. However, the irradiation of both cell types or the irradiation of tumor cells alone both led to increased adhesion. Thus, we were able to show for the first time in vitro how the adhesion of tumor cells to primary endothelial cells changes depending on the irradiation state of both cell types.

Cristofanilli et al. detected circulating tumor cells (CTCs) in peripheral blood of breast cancer patients and postulated a relationship between the amount of CTCs and a poor prognosis due to recurrence or metastasis formation [37, 38]. These CTCs were extremely aggressive, when injected into the mouse and were also able to colonize the primary tumor tissue as well as surviving and proliferating in distant organs. [14, 39]. However, the prerequisite for any new tumor formation from CTCs is that these leave the blood vessels via an adhesion process. So far, it is unclear whether and how radiotherapy affects the malignancy of tumor cells that have escaped local therapy. Our data show that the adhesion of circulating tumor cells to the endothelium is increased after irradiation. It was possible to show this both on a molecular and macroscopic level. After irradiation, not only was the expression of numerous adhesion proteins increased, but also the adhesion of tumor cells to an endothelial monolayer. This increased cell-cell adhesion was also observed in a pump system that experimentally mimics natural blood flow. Yuan et al. also showed in their work that a radiation dose of 5 Gy increased the expression of integrin α4 and integrin β1 on the membrane surface of murine monocytes/macrophages. Under flow conditions, they observed an increase in adhesion of macrophages to a VCAM-1 coated surface after 0.5 Gy irradiation. However, this decreased sharply with the radiation dose of 5 Gy, something which was not due to cell death. The authors attribute reduced adhesion after high doses to a regulatory function of reactive oxygen species (ROS) [40]. In contrast to these experiments with leukocytes, after irradiation with 4 Gy we found approximately 25 to 45% more tumor cells attached to the endothelial monolayer under “flow” conditions (Fig. 1d, e). This different adhesion behavior could be due to the fact that with VCAM-1 there is only one single binding possibility for the macrophages, whereas the surface of an endothelial cell offers several binding partners and thus a higher binding affinity between tumor cells and endothelial cells. In addition, leukocytes probably react much more sensitively to ionizing radiation than cancer cells.

The tumor cells adhere to endothelial cells via surface proteins that are expressed on both cell types. Here we examined the influence of ionizing radiation on the expression of various adhesion-associated proteins: *E-selectin, integrin α4, integrin α5, integrin β1, N-cadherin, CD44, ICAM-1 and VCAM-1*. After irradiation with 4 and 8 Gy, the expression of all of these proteins was significantly increased in endothelial cells (Fig. 2). As early as 20 years ago it was already described that ionizing radiation (1 Gy, 5 Gy and 10 Gy) upregulates the expression of adhesion molecules (ICAM-1, VCAM-1 and E-selectin) in normal endothelial cells and blood vessels, which led to increased adhesion of leukocytes [41–43]. We also observed an upregulation in the expression of about 60–70% of the investigated proteins in tumor cells. Other groups showed an increase in proteins of the integrin family (integrin α4, integrin α5 and integrin β1) in melanoma and breast cancer cell lines after irradiation, whereby the adhesion of these cells to fibronectin increased [44, 45]. Similarly to these studies, we found an increased expression of integrins in glioblastoma cells, breast cancer cells and endothelial cells after irradiation with 4 Gy or 8 Gy. An increased expression of these proteins was already measurable after 4 h and remained up to 24 h.

Treatment of glioblastoma cells with the FAK inhibitor PF-573, 228 reduced adhesion by almost 20%. In combination with a radiation dose of 4 Gy, the amount of attached tumor cells decreased by a further 5 to 10%. The adhesion of breast cancer cells MDA-MB-231 also decreased by about 50% after treatment with the inhibitor (Fig. 4). In fact, PF-573, 228 specifically inhibits the catalytic activity of FAK. It interacts with FAK through its ATP binding pocket and inhibits FAK autophosphorylation on tyrosine 397. This inhibitor also simultaneously reduces phosphorylation of paxillin and reorganizes the effectors of the FAK signaling pathway downstreams [27, 28, 33]. Our in vitro studies support these observations. In addition to increased expression of Src, Src-Tyr 416, FAK and paxillin, we observed strong phosphorylation of FAK on Tyr 397 in tumor cells after irradiation (Fig. 3), which is suppressed by the inhibitor (Fig. 4e, f) and thus also reduces adhesion. Activation of Src by irradiation has also been demonstrated in breast cancer cells, which was responsible for the invasive phenotype of the cells [14]. The role of FAK in the regulation of cell migration and adhesion, especially in integrin-mediated cell adhesion, has long been known [25]. Integrins are thought to be expressed more highly in different tumor entities than in normal healthy tissues [46] and their expression is thought to be increased by ionizing radiation [47]. Our experiments also show an increase in the expression of integrins after irradiation. Part of the regulation of cell adhesion via integrins is the autophosphorylation of FAK on tyrosine 397 and thus the formation of the FAK-Src complex, which then activates further kinases [25]. It is therefore conceivable that irradiation with photons increases both the expression and the activity of FAK and Src, which influences the expression of integrins and other adhesion proteins and hence adhesion. Some differences in the adhesion of glioblastoma cells and breast cancer cells on endothelial cells depending on which cell types have been irradiated could be observed, but this was not reflected in the expression of adhesion proteins after irradiation. However, increased cell adhesion and phosphorylation of FAK on tyrosine 397 was observed in both glioblastoma and breast cancer cells.

## Conclusions

Vilalta et al. show not only that irradiation increases the invasion of tumor cells, but also that irradiation of a tumor in a mouse model up to a certain dose of radiation stimulates the recruitment of circulating tumor cells [48]. The published data show that changes in the tumor cells as well as in the cells in the surrounding area occur as a result of irradiation. However, the molecular biological interrelationships and mechanisms of these effects have not been sufficiently investigated. Our work shows that ionizing radiation increases the adhesion of tumor cells to endothelial cells and the adhesion-associated surface proteins in both cell types and could thus possibly influence an important step in the metastasis cascade. However, since the clinical data do not clearly indicate an increased risk of metastasis due to radiation, we speculate that the success of radiotherapy and thus the prognosis for patients could be improved by combination with the FAK inhibitor (PF-573, 228).

## Abbreviations

CTC: Circulating tumor cell
EC: Endothelial cell
FAK: Fokal adhesion kinase
FAK-I: FAK-Inhibitor
HUVEC: Human umbilical vein endothelial cell
ROS: Reactive oxygen species
TC: Tumor cell
